# MOVING PICTURES: RAISING AWARENESS OF DEMENTIA IN CALD COMMUNITIES THROUGH MULTIMEDIA

**DOI:** 10.1093/geroni/igz038.1693

**Published:** 2019-11-08

**Authors:** Bianca Brijnath, Josefine Antoniades, Jon Adams, Collette Browning, Dianne Goeman, Katie Ellis, Mike Kent

**Affiliations:** 1 National Ageing Research Institution (NARI), Parkville, Victoria, Australia; 2 National Ageing Research Institute, Parkville, Victoria, Australia; 3 University of Technology Sydney, Ultimo, New South Wales, Australia; 4 Australian National University, Canberra, Australian Capital Territory, Australia; 5 University of Newcastle, Callaghan, New South Wales, Australia; 6 Curtin University, Bentley, Western Australia, Australia

## Abstract

Limited awareness of dementia in people from culturally and linguistically diverse (CALD) backgrounds often results in delayed diagnosis, poorer prognosis, and a higher burden of care on families and health systems. Given the rapidly ageing and multicultural populations in migrant-receiving countries such as Australia and the United States, this disparity needs to be addressed urgently. This project aimed to inform and educate people from five linguistically diverse backgrounds – Hindi, Tamil, Mandarin, Cantonese, and Arabic – about dementia. A mixed methods, multimedia design comprising video-interviews with 76 participants including carers from the five language groups and key service providers was employed. Data were gathered nationally across Australia in 2018 and thematically analysed. Data were used to co-produce 15 short films, comics, and a mobile-optimised website from which data analytics were measured. The films and comics focused on dementia detection and timely diagnosis, how to navigate the aged care system, and the importance of self-care. Analytics data is currently being collected online and via community forums. In conclusion, co-production methods in tandem with digital multimedia are fundamental to developing culturally salient interventions to address dementia disparities in CALD populations in Australia and internationally.

